# Feasibility, Usability, and Effects of Leisure-Based Cognitive Training Using a Fully Immersive Virtual Reality System in Older Adults: Single-Arm Pretest-Posttest Pilot Study

**DOI:** 10.2196/66673

**Published:** 2025-10-09

**Authors:** I-Ching Chuang, Xiao-Ting Huang, I-Chen Chen, Yih-Ru Wu, Ching-Yi Wu

**Affiliations:** 1 Department of Occupational Therapy and Graduate Institute of Behavioral Sciences, College of Medicine, Chang Gung University, No. 259 Wen-hwa First Road, Taoyuan, 33302, Taiwan, 886 032118800 ext 5761; 2 Department of Neurology, Linkou Chang Gung Memorial Hospital, Taoyuan, Taiwan; 3 Department of Occupational Therapy, College of Nursing and Health Sciences, Da-Yeh University, Changhua, Taiwan; 4 School of Medicine, Chang Gung University, Taoyuan, Taiwan; 5 Department of Physical Medicine and Rehabilitation, Linkou Chang Gung Memorial Hospital, Taoyuan, Taiwan; 6 Healthy Aging Research Center, Chang Gung University, Taoyuan, Taiwan

**Keywords:** cognitive training, leisure activities, feasibility, usability, immersive virtual reality, older adults

## Abstract

**Background:**

Cognitive training is an effective approach to support cognitive function in older adults. Incorporating meaningful leisure activities, such as gardening, may enhance both engagement and training outcomes. While fully immersive virtual reality (VR) offers ecologically valid and engaging environments that can further boost motivation, limited research has explored the combination of VR-based cognitive training and leisure activities for older adults.

**Objective:**

This study aims to assess the feasibility, usability, and preliminary effectiveness of leisure-based VR cognitive training for community-dwelling older adults.

**Methods:**

A fully immersive VR cognitive training system, controlled via a head-mounted display, was developed, incorporating gardening-themed activities such as planting, fertilizing, watering, and harvesting. These tasks were designed to engage multiple cognitive domains, including memory, attention, executive function, processing speed, and visuospatial abilities. The program consisted of 16 sessions delivered over 8 weeks (twice weekly, 1 hour per session). Cognitive outcomes were assessed before and after training using the Montreal Cognitive Assessment, the digit symbol substitution test, word list immediate and delayed recall, spatial span, and the Stroop Color and Word Test. Feasibility, acceptance, and usability were evaluated using the System Usability Scale and a posttraining questionnaire. Licensed occupational therapists from both community and institutional settings assessed the training system’s usability.

**Results:**

All 41 participants (mean age 69.79, SD 5.05 y) completed the training with 100% adherence and no serious adverse events. Feasibility ratings—particularly for perceived usefulness, intention to use, and subjective norms—reflected strong acceptance. Usability ratings from older adults indicated high ease of use, enjoyment, and positive experience, while professionals rated the system as moderately usable (mean System Usability Scale score 68.01, SD 8.38). Statistically significant improvements were observed in general cognition (*P*=.004), processing speed (*P*=.049), immediate and delayed memory (*P*<.001), and executive function (*P*=.002). No significant changes were found in visuospatial memory (*P*=.29).

**Conclusions:**

This study provides preliminary evidence supporting the feasibility and usability of a gardening-based VR cognitive training program for older adults. Feasibility was demonstrated through full adherence, absence of major adverse events, and high participant acceptance. Usability feedback was favorable from both older adults and professionals across community and long term care settings. Additionally, improvements in multiple cognitive domains, including general cognition, processing speed, memory, and executive function, suggest potential cognitive benefits. Future randomized controlled trials with more diverse samples and extended follow-up are warranted to confirm and expand upon these findings.

## Introduction

### Background

The rapid growth of the global aging population is expected to lead to an escalation of age-related cognitive decline, making it a noteworthy health concern. Cognitive decline may lead to dependence on others in engaging in daily activities [1], contributing to a growing need for social care [2]. Providing early preventive training to preserve and maintain cognitive functions is crucial for older adults in the community.

Cognitive training, involving the repetitive practice of standardized tasks [3], is grounded in the principles of neuroplasticity. Cognitive training often targets various cognitive domains, including attention, memory, visuospatial function, processing speed, and executive function [4,5]. The results of previous studies have shown that cognitive training has a small to moderate effect on attention, memory, visuospatial abilities, and executive function in older adults [5-8]. Despite the demonstrated effectiveness of cognitive training, its impact remains constrained [9].

Engaging in leisure activities, commonly perceived as enjoyable and easily accessible, may provide protective effects against age-related cognitive decline and dementia [10]. Integrating leisure activities into cognitive training has the potential for enhancing the effectiveness of the enjoyment and meaningfulness of interventions for older adults, with relatively straightforward implementation in community settings [11]. Horticultural therapy is one of the favorite leisure activities for older adults because gardening-related activities resonate with their past experiences [12]. Growing evidence supports the cognitive benefits of gardening in older adults [13-17], as demonstrated in both systematic reviews and empirical studies showing improvements in memory, attention, and overall cognitive function, alongside enhanced physical health and well-being [16,17]. Physiologically, gardening has been associated with increased levels of brain-derived neurotrophic factor and platelet-derived growth factor, which are linked to neurogenesis and memory enhancement [13,14]. Furthermore, large-scale population-based and intervention studies suggest that gardening may help reduce the risk of cognitive decline and improve cognitive performance, including in individuals with mild to moderate dementia [15,16]. The integration of horticultural activities into cognitive training may be a strategy for enhancing cognitive function in older adults.

Fully immersive virtual reality (VR) augments ecological validity, provides immediate performance feedback, and facilitates customization of environments and activities, thereby conferring advantages over conventional cognitive training [18]. Feasibility studies on cognitive training using VR technology have demonstrated that older adults accepted and were motivated by VR-based tasks [19-21]. VR cognitive training has been effective in enhancing cognitive function in older adults, including global cognition, visuospatial processing, visual memory, and executive function, both in individuals with and without cognitive decline [22-27]. Recent studies have shown that immersive VR significantly enhances cognitive performance in individuals with mild cognitive impairment or dementia, particularly in executive function and global cognition [28]. Randomized trials also indicate that VR-based cognitive-motor training yields cognitive improvements comparable to traditional approaches, with the added benefits of greater task engagement and user interaction [29]. Furthermore, immersive simulations of daily tasks can stimulate multiple cognitive domains such as attention, memory, and planning [30]. These findings underscore the potential of immersive VR as an effective and practical cognitive training tool for older adults. A recent review also highlights that relevance of content, ease of use, and user support are key to successful implementation in aged care settings [31].

Most previous studies of immersive VR cognitive training for older adults have focused on daily activities [21,24,25,32] or, for convenience, have used commercially available gaming scenarios, such as Jet Run [33], rather than designing serious gaming activities [22]. This might easily result in older adults perceiving the training process as less enjoyable and meaningful, thereby limiting the effectiveness of the cognitive training. Incorporating leisure activities into immersive VR cognitive training could enhance the enjoyment and meaningfulness of the training for older adults, leading to an increase in its effectiveness [9]. However, studies on incorporating leisure activities into immersive VR cognitive training for older adults remain limited.

Conducting feasibility and usability testing for a VR training program is considered a prerequisite for its successful implementation [34]. For the implementation of VR training in older adults, the significance of usability is underscored by age-related factors that contribute to barriers in use [34]. Consequently, considering the perspective of professionals may improve the applicability of VR training for older adults [35]. However, few studies have comprehensively investigated the usability of VR cognitive training for professionals.

### Objectives

This study aims to investigate the feasibility and usability of leisure-based cognitive training using a VR system for community-dwelling older adults and evaluate its preliminary effectiveness. We hypothesize that leisure-based cognitive training with VR would be feasible, acceptable, and enjoyable for older adults. We also expect a good acceptance of the VR system. On the basis of the reviewed literature, we further hypothesize that leisure-based VR cognitive training may enhance cognitive domains such as global cognition, memory, and executive function in older adults [22,23,25,27].

## Methods

### Participants

The inclusion criteria were (1) age ≥60 years, (2) a Montreal Cognitive Assessment (MoCA) score of ≥21, and (3) the ability to follow instructions. A MoCA threshold of 21 was selected to include older adults with mild cognitive decline who retained sufficient cognitive capacity for interacting with VR systems, while avoiding the exclusion of those with subtle impairments who may still benefit from feasibility testing. Exclusion criteria included a diagnosis of dementia or other major psychiatric disorders, a history of motion sickness, or psychosis-related symptoms that could interfere with VR training.

Demographic information, including education, age, and sex, was collected using a structured questionnaire at the time of participant enrollment. Data were gathered through face-to-face interviews after obtaining participant consent.

In addition to the older adult participants, 40 licensed occupational therapists (n=20, 50% from day care centers or long-term care homes and n=20, 50% from community centers) were recruited to evaluate the usability of the VR cognitive training system. These professionals had at least one year of relevant clinical experience. Owing to their backgrounds, they were familiar with individuals both with and without mild or severe cognitive impairments and were therefore well positioned to assess usability across older adults with varying cognitive profiles.

### Sample Size Estimation

This pilot study included 41 participants, a sample size determined based on recruitment feasibility and literature guidelines. Prior studies suggest that 24 to 55 participants are adequate for exploratory trials [36,37], and a review reported a median of 30 participants in feasibility studies conducted in the United Kingdom [38].

### Materials

**Figure 1.**
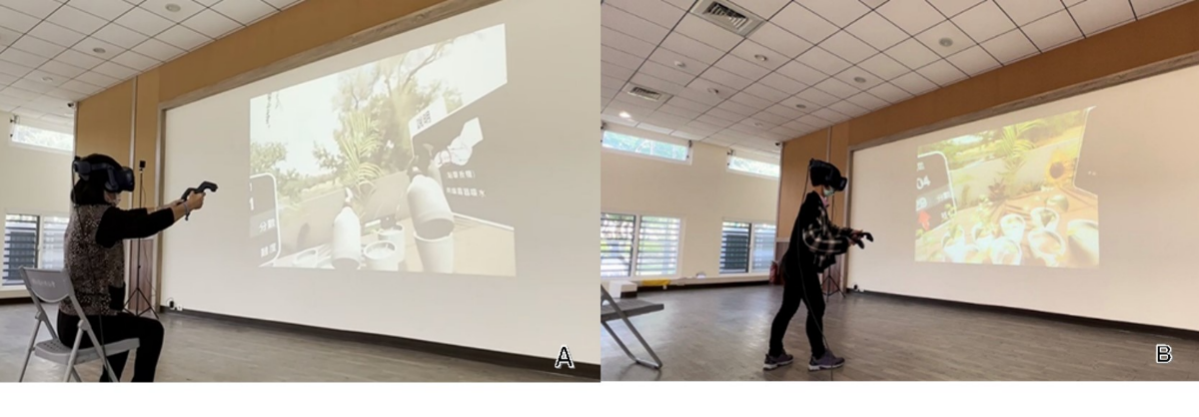
Community-dwelling older adults interacting with the immersive virtual reality cognitive training system during the intervention period. (A) Participant using the system in a seated position. (B) Participant using the system in a standing position.

**Figure 2.**
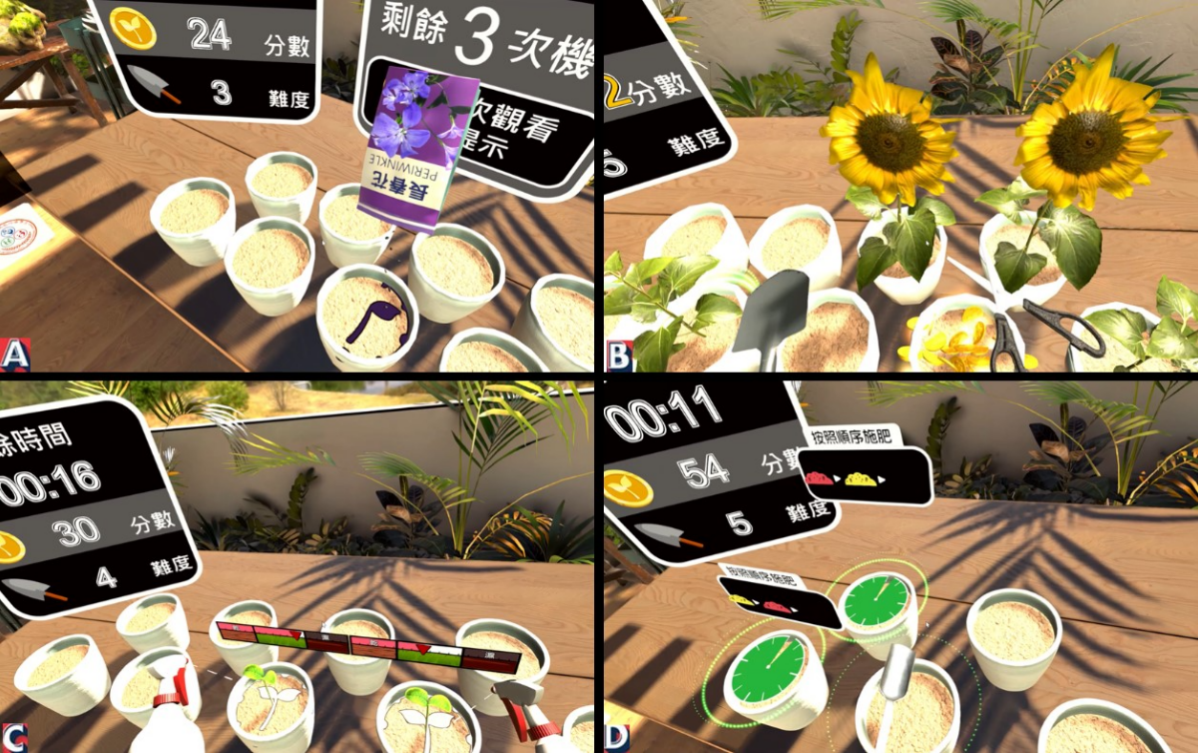
Screenshots from the immersive virtual reality cognitive training system showing examples of 4 gardening-related tasks used in the intervention: (A) planting, (B) harvesting, (C) watering, and (D) fertilizing.

The VR content was custom designed using Unity 3D software (Unity Technologies) and integrated with the HTC Vive Pro head-mounted display. The system supported full-body interaction using handheld controllers, allowing participants to perform tasks in either a seated or standing position depending on their physical condition (Figure 1). The training content featured realistic horticultural tasks with embedded cognitive demands and provided immediate visual and auditory feedback. The VR-based cognitive training program comprised more than 10 gardening-themed activities designed to engage multiple cognitive domains (Figure 2). Each gardening activity was designed to target specific cognitive domains based on its task demands; for example, the sowing task required participants to recall the color and position of illuminated flowerpots and match them with corresponding seed packets, thereby engaging working memory. The harvesting task involved monitoring multiple flowerpots for signs of blooming or wilting, selecting appropriate tools, and executing timely actions to either harvest blooming flowers or remove wilted plants, thereby training executive function. The watering task, in which participants monitored plant hydration levels and adjusted watering accordingly, targeted attention and processing speed. The fertilizing task required recalling and applying a sequence of fertilizer colors to the correct plants, enhancing working memory. These activities collectively supported the training of attention, memory, executive function, processing speed, and visuospatial abilities in an ecologically meaningful and engaging context. In each training session, at least 2 different cognitive domains were addressed to ensure comprehensive cognitive training throughout the program.

### Procedure

The VR cognitive training program consisted of 16 sessions delivered over 8 weeks (twice weekly, 1 hour per session). In previous studies on the application of VR cognitive training for older adults, the average number of training sessions was approximately 17, with each session lasting between 20 and 60 minutes [30]. The total training duration per participant was approximately 13 hours. Moreover, some studies adopted a training frequency of 2 to 3 sessions per week [25,39,40]. Therefore, we adopted a training schedule of 2 sessions per week, each lasting 1 hour, over an 8-week period. Each session consisted of 4 to 5 training segments, interspersed with breaks to prevent motion sickness. Training was guided by occupational therapists who received standardized instruction to ensure fidelity and consistency. Each therapist had more than two years of experience providing cognitive interventions for older adults in community or day care centers, enabling them to adjust the training based on participants’ responses as needed. When older adults had difficulty understanding the operations or tasks in the early stages of training, the therapists provided verbal and physical assistance. The data collection process was conducted by trained research assistants under the supervision of the principal investigator. Prior to data collection, all research assistants received standardized training to ensure consistency in administering both questionnaires and assessments. Data were collected between 2021 and 2022. To maintain procedural fidelity across sites, uniform protocols were implemented throughout the study.

### Outcome Measures

#### Feasibility Measures

Feasibility was evaluated through adherence (ie, attendance and completion rates), adverse events (eg, dizziness or discomfort), and participant acceptance of the VR training. Adherence was evaluated based on the attendance rate of older adults, defined as the number of participants who completed the programs divided by the number of participants at baseline. Therapists were required to report any adverse events related to the use of the VR system, such as headaches, head fullness, blurred vision, dizziness, and vertigo, that occurred during the study. To assess acceptance, participants were asked to complete selected items from an acceptance questionnaire (Multimedia Appendix 1) based on constructs from the unified theory of acceptance and use of technology [41] and the technology acceptance model [42]. The following subscales were used: perceived usefulness, subjective norms, and intention to use. These reflect the feasibility dimensions of acceptability and adoption potential [41,43]. Participants rated all items on a 5-point Likert scale ranging from 1=strongly disagree to 5=strongly agree [44].

#### Usability Measures

Usability was assessed from the perspectives of both participants and professionals. Participants completed the following additional subscales from the same acceptance questionnaire, focusing on user-system interaction: perceived ease of use, perceived enjoyment, and user experience. These dimensions align with the ISO (International Organization for Standardization) 9241‐11 criteria (effectiveness, efficiency, and satisfaction) and the usability heuristics formulated by Nielsen [45]. Participants rated these items on a 5-point Likert scale ranging from 1=strongly disagree to 5=strongly agree.

Professionals evaluated usability using the System Usability Scale (SUS; Multimedia Appendix 2) [46] after a single VR session. The SUS consists of 10 questions. Professionals rated each question on a scale ranging from 1=strong disagreement to 5=strong agreement. These ratings were then processed through an algorithm to produce a score out of 100 points.

#### Cognitive Assessments

For cognitive function, assessors conducted all outcome assessments before and immediately after the training. The reliable and valid assessments used included the MoCA [47,48], the digit symbol substitution test (DSST) from the Wechsler Adult Intelligence Scale [49-51], word list immediate and delayed recall from the Wechsler Memory Scale–Third Edition (WMS-III) [52,53], and the Stroop Color Word Test (SCWT) [54,55].

The MoCA is a widely used tool for assessing global cognitive function and is known for its high sensitivity in detecting cognitive decline [47]. The MoCA had high internal consistency (Cronbach α=0.86) [48]. Global cognitive functioning encompasses a range of cognitive abilities, such as orientation, attention, memory, naming, visuospatial skills, executive function, language, and abstraction. The assessment yields a total score of 30 points, with higher scores indicating superior global cognitive function.

The DSST, a subtest from the Wechsler Adult Intelligence Scale, measures information processing speed [49]. During the test, participants are required to match symbols to corresponding numbers and replicate these symbols in designated spaces within 120 seconds. The number of correctly completed symbols determines the DSST score. This test demonstrates high test-retest reliability [50] and strong sensitivity to changes in cognitive function [51]. Higher DSST scores reflect superior information processing speed.

The word list subtest from the WMS-III is a widely used tool for assessing working memory. The word list test comprises 2 parts: word list immediate recall and word list delayed recall. In word list immediate recall, participants are presented with 12 unrelated words over 4 trials and asked to recall them immediately after each presentation. Word list delayed recall asks participants to recall the same 12 words again 25 to 35 minutes after completing word list immediate recall. Higher word list immediate recall or word list delayed recall scores signify better immediate or delayed recall, respectively. The spatial span subtest from the WMS-III is used to assess visuospatial memory. During the spatial span subtest, participants are instructed to touch sequentially positioned blocks in either the same order as demonstrated by the instructor or in reverse order. The word list and spatial span subtests from the WMS-III demonstrate good internal consistency, with reported Cronbach α coefficients ranging from 0.78 to 0.94 [52]. The reliability and validity of the WMS-III have been established in community-dwelling adults, making it a valid tool for assessing cognitive improvements after training [53].

The SCWT is a widely used assessment of inhibitory executive function. Its internal consistency has been demonstrated to be reliable, with Cronbach α coefficients averaging 0.80 [54]. The SCWT is sensitive in distinguishing individuals with cognitive decline from those without [55]. Performance differences between congruent and incongruent tasks indicate response interference. We measured inhibitory executive function by calculating the time differences between the congruent and incongruent tasks, with higher SCWT scores indicating poorer inhibitory executive function.

### Ethical Considerations

This study was approved by the institutional review board of Chang Gung Memorial Hospital (202100553B0). All participants provided written informed consent prior to enrollment. To protect participants’ privacy and confidentiality, all data were deidentified prior to analysis. Participants received a gift valued at approximately NT $50 (US $1.65) as a token of appreciation. No identifiable images of participants are included in the manuscript.

### Statistical Analysis

The demographic characteristics of the participants are reported using descriptive statistics, including means and SDs. A paired 2-tailed *t* test was conducted to evaluate the effect of the leisure-based VR cognitive training program. The significance level was set at .05 for all statistical tests. SPSS software (version 27.0; IBM Corp) was used for data analysis.

## Results

### Participant Characteristics

The study recruited 41 older adults from local community facilities. Participants ranged in age from 62 to 82 (mean 69.79, SD 5.05) years. Of the 41 participants, 6 (15%) were male, and 35 (85%) were female. They had an average of 12.11 (SD 3.80) years of education. The participants’ demographic data and baseline characteristics are presented in Table 1.

Most of the participants experienced no adverse events during the VR training, except for 1 older adult who reported experiencing dizziness once during the middle phase of the training, which resolved after a brief period of rest. All 41 older adults completed the 16-session program, yielding a 100% adherence rate.

**Table 1.** Demographic data and baseline characteristics of community-dwelling older adults participating in the virtual reality cognitive training program. Attendance was 100%, with all older adults completing the 16 sessions of the program (N=41).

Characteristics	Participants
Age (years), mean (SD)	69.79 (5.05)
Sex, n (%)	
Male	6 (15)
Female	35 (85)
Education (years), mean (SD)	12.11 (3.80)
Montreal Cognitive Assessment score, mean (SD)	27.82 (2.44)

### Intervention Acceptability

Regarding intervention acceptability, older adult participants reported high scores on items reflecting perceived usefulness, subjective norms, and intention to use, with a mean feasibility score of 4.11 (SD 0.61) on a 5-point Likert scale. Notably, perceived usefulness and intention to use both exceeded a mean of 4, suggesting strong acceptance and adoption potential (Table 2).

Older adult participants reported favorable experiences regarding system usability. Subscale scores for perceived ease of use, enjoyment, and user experience yielded a mean usability score of 4.23 (SD 0.58) on a 5-point Likert scale, indicating that the system was generally perceived as intuitive, enjoyable, and user-friendly (Table 2).

**Table 2.** Results of the posttraining acceptance questionnaire assessing the feasibility and usability of the virtual reality cognitive training experience from the perspective of older adults (N=41).

Variables	Scores, mean (SD)
Feasibility subscale	
Perceived usefulness	4.42 (0.49)
Intention to use	4.34 (0.59)
Subjective norms	3.57 (0.73)
Usability subscale	
Perceived ease of use	3.93 (0.65)
Perceived enjoyment	4.34 (0.58)
User experience	4.42 (0.49)

From the professionals’ perspective, usability was evaluated using the SUS. A total of 40 occupational therapists participated in the evaluation (n=20, 50% from day care centers or long-term care homes and n=20, 50% from community centers). The overall SUS mean score was 68.01 (SD 8.38), indicating an acceptable level of usability [56]. An independent samples *t* test was conducted to compare SUS scores between professionals from day care centers or long-term care homes and those from community centers. No significant difference was found between the 2 groups (*t*_38_=0.78; *P*=.45), with institution-based professionals reporting a mean score of 69.63 (SD 1.83) and community-based professionals a mean score of 66.40 (SD 8.44).

After 16 training sessions, older adult participants showed significant improvements in the MoCA scores (Cohen *d*=0.47 [medium effect]; *P*=.004), DSST (Cohen *d*=0.16 [small effect]; *P*=.049), word list immediate recall (Cohen *d*=0.58 [medium effect]; *P*<.001), word list delayed recall (Cohen *d*=0.39 [small to medium effect]; *P*<.001), and the SCWT (Cohen *d*=0.41 [medium effect]; *P*=.002). No significant pretest-posttest differences were found for the spatial span (Cohen *d*=0.15 [small effect]; *P*=.29; Table 3).

**Table 3.** Results of paired sample *t *tests, with mean (SD) values, for pretest-posttest outcome measures in older adults (N=41).

Outcome measures	Baseline scores, mean (SD)	Posttraining scores, mean (SD)	*t* test (*df*)	*P* value	Effect size (Cohen *d*)
MoCA^a^	27.76 (2.55)	28.76 (1.55)	3.03 (40)	.004	0.47
DSST^b^	62.02 (15.48)	64.54 (15.27)	1.05 (40)	.049	0.16
Wechsler Memory Scale–Third Edition					
Word list immediate recall	29.76 (7.33)	34.10 (7.64)	3.71 (40)	<.001	0.58
Word list delayed recall	12.73 (3.0)	13.93 (3.14)	2.5 (40)	<.001	0.39
Spatial span	14.24 (3.02)	14.71 (3.32)	−2.65 (40)	.29	0.15
SCWT^c^	16.55 (8.62)	13.39 (6.52)	0.95 (40)	.002	0.41

a MoCA: Montreal Cognitive Assessment.

b DSST: digit symbol substitution test.

c SCWT: Stroop Color Word Test.

## Discussion

### Principal Findings

To the best of our knowledge, this is the first study to examine the feasibility, usability, and preliminary cognitive outcomes of a fully immersive VR cognitive training system incorporating gardening-themed leisure activities for community-dwelling older adults. The findings support the feasibility of the intervention, as evidenced by full attendance, minimal adverse events, and high levels of acceptance reflected in participants’ responses to perceived usefulness, subjective norms, and intention to use. Both older adults and health professionals provided favorable impressions of the system’s usability. Among older adults, high ratings on perceived ease of use, perceived enjoyment, and user experience indicated that the system was intuitive and engaging. Likewise, professionals from both community and institutional settings rated the system as acceptably usable based on the SUS. Moreover, the 8-week program led to significant improvements in general cognition, processing speed, immediate recall, delayed recall, and executive function. These findings support the notion that incorporating meaningful leisure activities, such as gardening, into VR-based training offers an engaging and ecologically valid strategy to enhance cognitive function among community-dwelling older adults.

### Feasibility Perspectives Among Older Adults

Feasibility was supported by excellent adherence (100% program completion) and minimal adverse events, consistent with prior VR studies involving older adults [24,57]. Participant acceptance of the VR system was high, particularly in terms of perceived usefulness and intention to use, with both averaging above 4 on a 5-point Likert scale. These results suggest that the leisure-based and culturally familiar content helped foster engagement and adoption potential. The relatively lower score in subjective norms may reflect neutral or uncertain perceptions among family members or caregivers regarding the necessity of VR training, which aligns with previous research noting that social influence is often less pronounced in early-stage adoption [41]. Nevertheless, the overall acceptance profile indicates strong feasibility for broader implementation in community settings.

### Usability Perspectives From Older Adults and Professionals

The usability of the leisure-based VR cognitive training system was supported by both older adult participants and professionals from both community and institutional settings. Older adults reported high scores across usability-related domains, including perceived ease of use, perceived enjoyment, and user experience, indicating that the system was generally intuitive, engaging, and user-friendly. These findings suggest that the training interface and task flow were well suited for older users, even among those with limited prior exposure to immersive technologies.

From the professionals’ perspective, system usability was evaluated using the SUS, which yielded an average score indicative of acceptable usability [58]. Ratings were generally positive, with no significant differences observed between professionals working in day care centers or long-term care homes and those working in community centers. Despite minor variations in mean scores, the results suggest that the system was perceived as similarly usable across settings. This consistency underscores the flexibility and applicability of the VR training system in both long-term care homes and community-based environments.

### Effects on Cognitive Function

Our study demonstrated that the 16-session leisure-based VR cognitive training program led to improvements in general cognition, processing speed, immediate and delayed recall, and executive function in older adults. These findings extend previous research on VR showing significant enhancements in general cognition, executive function, and memory [59,60], suggesting that leisure-based activities, especially gardening, might improve various aspects of cognitive ability. Gardening procedures such as sowing seeds, deciding the amount of watering, and making embossed flowers require different cognitive abilities to complete the task successfully. Practicing such tasks might directly benefit cognitive performance. Effect sizes ranged from small to moderate, reflecting varying impacts of the training on cognitive outcomes.

Our results are consistent with earlier studies demonstrating the cognitive benefits of VR interventions for older adults [59,60]. The incorporation of gardening elements, such as sequencing planting tasks, adjusting watering levels, and managing virtual tools, appears to offer additional ecological validity and engagement. However, our findings diverged from previous work in visuospatial memory [60], which did not improve significantly. One possible explanation is that our VR training primarily emphasized object-to-object spatial relations. The assessment and management of self-to-object spatial relations in spatial relationships typically require enhanced perceptual and adjustment abilities from individuals and may therefore be more challenging than assessing and managing object-to-object spatial relations [61]. As a result, no significant enhancement was observed in visuospatial memory.

### Strengths and Limitations

This study has several strengths, including the development of a fully interactive and task-oriented VR system, excellent adherence (100% program completion), and the integration of feedback from professionals across diverse care settings.

However, several limitations should be noted. First, the participants were community-dwelling older adults who regularly engaged in community activities. These individuals were likely more motivated and cognitively active, which may have contributed to more favorable training outcomes. This relatively homogeneous and proactive sample may limit the generalizability of the findings to the broader population of older adults, particularly those with lower levels of engagement or residing in institutional settings. Future studies may aim to recruit a more diverse population to enhance the external validity and applicability of the results across various subgroups of older adults.

Second, the history of motion sickness in older adults was assessed via self-report rather than using a standardized questionnaire. Although therapists continuously monitored and documented adverse events related to the use of the VR system during training, the study did not include a formal assessment of the incidence of cybersickness resulting from VR exposure. Future research could address these limitations by incorporating standardized tools to assess both the history of motion sickness and the occurrence of cybersickness, thereby enhancing the accuracy and reliability of the findings.

Third, participants were informally asked about their gardening experience—approximately one-third reported prior gardening activity—but no formal data were collected regarding their familiarity with gardening tasks or prior use of VR technologies. The lack of detailed assessment of prior experience with gardening or technology may have influenced participants’ engagement and performance during the training. Future studies might consider systematically capturing such background characteristics to better interpret individual differences in outcomes.

Fourth, the absence of a control group limits the ability to attribute the observed cognitive and functional improvements solely to the VR-based intervention. Without a comparison group, it is challenging to rule out the influence of confounding variables such as spontaneous recovery, learning effects, or engagement in external training activities. Furthermore, this study could not determine whether VR training outperforms traditional cognitive training. To validate the findings of this study and demonstrate the possible superiority of VR training, future studies may use a randomized controlled trial design with different control groups, including an active control group (eg, traditional cognitive training), a passive control group (eg, watching leisure videos), and a no-intervention group.

Finally, this study only examined immediate pretest-posttest intervention effects. Without a follow-up assessment, it is not possible to determine the long-term sustainability of the observed cognitive improvements. Future studies may incorporate longitudinal follow-up assessments to determine whether the benefits of VR-based cognitive training are sustained over time and to further explore its potential for promoting long-term cognitive improvement.

In summary, addressing these limitations through rigorous study designs, including randomized controlled trials, standardized tools to assess cybersickness, detailed participant profiling, and longitudinal tracking, will strengthen the evidence base and clarify the potential of VR-based leisure training for cognitive function among community-dwelling older adults.

### Conclusions

We developed a gardening-based, leisure-oriented cognitive training program using a VR system tailored for older adults in community settings. Our findings support the feasibility of this program, as demonstrated by full participant adherence, the absence of major adverse events, and high levels of acceptance. Older adults perceived the program as useful, expressed willingness to use it in the future, and felt supported by others. Usability ratings were also favorable, with both older adults and professionals in the community and in long-term care homes reporting positive user experiences. Preliminary cognitive outcomes showed statistically significant improvements in general cognition, processing speed, immediate and delayed recall, and executive function. However, due to the absence of a control group and the limited sample size, causal inferences cannot be drawn. These findings should be interpreted as initial evidence of potential benefit. Future research using randomized controlled trial designs, long-term follow-up assessments, and more diverse participant groups is needed to validate and extend these results.

## Supplementary material

10.2196/66673 Multimedia Appendix 1 Questionnaire: acceptance of the virtual reality experience among older adults.

10.2196/66673 Multimedia Appendix 2 The System Usability Scale [46].

## Abbreviations

DSST: digit symbol substitution test
ISO: International Organization for Standardization
MoCA: Montreal Cognitive Assessment
SCWT: Stroop Color Word Test
SUS: System Usability Scale
VR: virtual reality
WMS-III: Wechsler Memory Scale–Third Edition
