# Supplementary material for: Feasibility, Usability, and Effects of Leisure-Based Cognitive Training Using a Fully Immersive Virtual Reality System in Older Adults: Single-Arm Pretest-Posttest Pilot Study
Source: JMIR Serious Games. 2025 Oct 9;13:e66673. doi: 10.2196/66673 (PMC12510613; doi:10.2196/66673)
Supplement: Multimedia Appendix 2 [file games-v13-e66673-s002.docx]

The System Usability Scale (SUS)

| This survey is designed to understand the perceptions and evaluations of older adults in your community or facility regarding the usability of virtual reality cognitive training (hereafter referred to as VR training). Please read each statement carefully and check the box on the right that best matches your answer. There are no right or wrong answers. Please answer quickly and honestly, without spending too much time on any one question. |
| --- |

| Items | strongly  disagree  1 | 2 | 3 | 4 | strongly  agree  5 |
| --- | --- | --- | --- | --- | --- |
| 1. I think that older adults would like to use this system frequently. | □ | □ | □ | □ | □ |
| 2. I found the system unnecessarily complex to older adults. | □ | □ | □ | □ | □ |
| 3. I thought the system was easy to use for older adults. | □ | □ | □ | □ | □ |
| 4. I think that older adults would need the support of a technical person to be able to use this system. | □ | □ | □ | □ | □ |
| 5. I found the various functions in this system were well integrated. | □ | □ | □ | □ | □ |
| 6. I thought there was too much inconsistency in this system. | □ | □ | □ | □ | □ |
| 7. I would imagine that most older adults would learn to use this system very quickly. | □ | □ | □ | □ | □ |
| 8. I found the system very cumbersome for older adults to use. | □ | □ | □ | □ | □ |
| 9. I felt older adults were very confident in using the system. | □ | □ | □ | □ | □ |
| 10. I think older adults need to learn a lot of things before they can get started with this system | □ | □ | □ | □ | □ |

The System Usability Scale (SUS), developed by John Brooke in 1986, is publicly available and free to use without license, provided the original source is cited.

Brooke J. SUS: a quick and dirty usability scale. In: Jordan PW, Thomas PW, Weerdmeester BA, McClelland IL, editors. Usability Evaluation in Industry. Taylor & Francis; 1996:189-194.
